# Biological characterization and *in vitro* fungicide screening of a new causal agent of walnut leaf spot in Guizhou Province, China

**DOI:** 10.3389/fmicb.2024.1439487

**Published:** 2024-10-09

**Authors:** Xianxi Ao, Ting Shi, Wenjia Yang, Hao Ouyang, Ruidong Fan, Junaid Ali Siddiqui, Chaoming Wu, Zhoule Lv, Shasha Deng, Xiaoyulong Chen

**Affiliations:** ^1^Key Laboratory of Surveillance and Management of Invasive Alien Species in Guizhou Education Department, College of Biological and Environmental Engineering, Guiyang University, Guiyang, China; ^2^College of Agriculture/College of Life Sciences, Guizhou University, Guiyang, China

**Keywords:** *Juglans regia*, leaf spot, *Didymella segeticola*, biological characterization, fungicides screening

## Abstract

Walnut (*Juglans regia* L.) is a widely grown nut plant worldwide, including in Guizhou Province, located in southwest China. The high quality and special taste make Guizhou walnuts, particularly those produced in Hezhang County, a “Chinese National Geographical Indication Product” that substantially contributes to the local economy and grower’s income. In July 2022, a serious occurrence of leaf spot disease was observed in a walnut plantation area, Shuitang Town, Hezhang County, Guizhou Province, China (27°07′67″N, 104°64′61″E). The causal agent was identified as *Didymella segeticola* through morphological characterization and amplification and sequencing of the internal transcribed spacer (ITS) region, beta-tubulin (*TUB*) gene, and glyceraldehyde-3-phosphate dehydrogenase (*G3PD*) gene. Koch’s postulates, including re-isolation and identification, were performed to confirm its pathogenicity on healthy leaves. To our knowledge, this is the first report of *D. segeticola* causing leaf spot on walnuts worldwide. Further, to determine its biological characteristics, which could be utilized for future disease management, the effects of temperature, light, and carbon and nitrogen resources on mycelial growth, conidia production, and conidia germination and the effects of humidity on conidia germination were studied. The optimum temperature for mycelial growth of representative strain *D. segeticola* C27 was 20°C. Increasing the light period significantly decreased conidia production and conidia germination. Maltose and beef extract were the best carbon and nitrogen sources, respectively, for the pathogen. Conidia germination was enhanced at 90% humidity. *In vitro* screening of effective fungicides was conducted. Among the 20 screened fungicides, difenoconazole showed the best inhibition rate, with an EC_50_ (concentration for 50% of the maximal effect) of 0.0007 μg/mL. Tetramycin also showed sufficient inhibitory effects against *D. segeticola*, with an EC_50_ value of 0.0009 μg/mL. Our study provides new insights into the causal agent of walnut leaf spot in Guizhou, China, as well as the first pathogen characteristics and promising candidate fungicides for its management.

## 1 Introduction

Walnut (*Juglans regia* L.), a widely distributed and important cultivated nut plant acclaimed for its timber and nutritious kernels (Bayazit et al., 2007), is among the four most consumed dried fruits globally (Fordos et al., 2023). The history of walnut traces back to Persia around 7,000 BC (Dreher et al., 1996), and presently, it is commercially cultivated across Southern Europe, North Africa, East Asia, the United States, and western South America. Notably, China has emerged as the world’s leading producer of walnuts (Vahdati et al., 2019). The widespread appreciation of walnuts stems from their popularity and extensive consumption. Prior studies underscore their role in reducing the risk of heart disease caused by polyunsaturated fatty acids (Tapsell et al., 2004; Blomhoff et al., 2006; Hama and Fitzsimmons-Thoss, 2022; Glenn et al., 2023). Moreover, the roots, barks, leaves, and green core-shell peaches of walnuts have versatile utility, for example, in the pharmaceutical and cosmetic industries (Salejda et al., 2016). As such, all of the parts of the walnut tree are recognized as rich sources of diverse chemicals that contribute to the synthesizing of compound drugs. These drugs exhibit multifaceted therapeutic properties ranging from pain relief, anti-ulcer, anti-asthma, immune regulation, and larvicidal effects, thus positively influencing human health (Jahanban-Esfahlan et al., 2019). Hence, it is imperative to cultivate and protect the walnut industry sustainably (Sagawa et al., 2020).

However, the growth of *Juglans regia* L. faces significant threats from plant diseases, prim caused by bacteria, viruses, and fungi. For example, walnut bark canker caused by *Brenneria nigrifluens* (Charkhabi et al., 2010; Razinataj et al., 2020) and bacterial black spot induced by *Xanthomonas campestris* and *Pantoea agglomerans* have been found in Gansu, China (Wang et al., 2016). Similarly, walnut fruit canker caused by *Pseudomonas syringae* pv. *Syringae* has been reported in Iran (Keshtkar et al., 2016; Ahmadi et al., 2017). Walnut bacterial wilt caused by *Xanthomonas arboricola pv. Juglandis* (Xaj) has been reported in Korea (Kim et al., 2021). Notably, the emergence of the Plum pox virus infecting walnut trees was first documented in 1996 (Baumgartnerová, 1996), while a walnut disease associated by the Cherry leaf roll virus (CLRV) was found in Turkey in 2008 (Ozturk et al., 2008). Further, numerous fungal infections plague walnut trees and diminish fruit quality and yield. For example, in 2013, Chen et al. (2013) reported *Lasiodiplodia citricola* and *Neoscytalidium dimidiatum* as pathogens responsible for the decrease number of walnut trees. Subsequently, walnut anthracnose caused by *Colletotrichum siamense* led to severe damage to yield and quality of walnuts in Shandong Province, China, in 2017 (Wang et al., 2017), while the discovery of branch canker caused by *Diaporthe amygdali* in 2018 further adversely affected walnut cultivation in Shandong Province (Meng et al., 2018).

In this study, we investigated walnut cultivation in Bijie City, Guizhou Province, China, and we revealed the high incidence of walnut leaf spot, especially during the rainy season from June to August. This conducive environment facilitated the rapid proliferation and dissemination of the pathogen, resulting in widespread leaf spots on walnut leaves. Notably, walnut leaf spot can be caused by various pathogenic fungi. Prior studies have attributed walnut leaf spot to *Boeremia exigua* infection (Cai et al., 2021; Wang et al., 2022). In addition, Xu et al. (2023) reported walnut leaf spot disease caused by *Ragnhildiana diffusa* in Sichuan province of China, with symptoms of initially brown lesions encircled by yellow halos, progressing to extensive leaf coverage and eventual leaf shedding. Similarly, Wang F. H. et al. (2023) identified a leaf spot disease of walnut caused by *Nothophoma quercina* in Sichuan Province of China. Currently, fungicides are widely screened and utilized for walnut disease control. Prior studies have highlighted the efficacy of flusilazole, fludioxonil, propiconazole, and pyraclostrobin in inhibiting *Colletotrichum fioriniae* mycelial growth and spore germination, thus effectively preventing and managing walnut disease (Fan R. D. et al., 2023). Further, He et al. (2019) demonstrated the high sensitivity of *Colletotrichum gloeosporioides* s.s. to pyraclostrobin, difenoconazole, fipronil, tebuconazole, pyrazole, and tetramycin, inhibiting mycelial growth. In addition, Zhou et al. (2023) reported the effectiveness of prochloraz in controlling the leaf spot of *Carya cathayensis* caused by *Fusarium oxysporum*, *Botrytis japonica*, and *Botrytis cinerea*. Nevertheless, research on walnut leaf spot control remains limited. Prolonged reliance on chemical sterilization can foster pathogen resistance to fungicides (Tang et al., 2022; Li et al., 2023), as evidenced by prior studies demonstrating drug resistance among walnut pathogens (Wang et al., 2020).

Hence, screening chemical pesticides that exhibit low toxicity and high efficiency is paramount for disease control. In addition, comprehending the biology of pathogenic fungal is crucial to shed light on disease development and the emergence of fungicide resistance. Therefore, exploring the biology of these pathogens is indispensable. Accordingly, the objectives of this study were to (1) isolate and characterize the pathogens responsible for severe walnut leaf spot disease in the Hezhang County area of Bijie City, Guizhou Province, China; (2) elucidate the biological characteristics of pathogenic fungal; and (3) screen for effective fungicides to mitigate disease impact.

## 2 Materials and methods

### 2.1 Sample collection and fungal isolation

In July 2022, a severe outbreak of walnut leaf blight was identified in Shuitang Town, Hezhang County, Guizhou Province, China (27°07′67″N, 104°64′61″E). Within an evaluation area spanning 0.8 hectares, disease incidence rates reached 65–75%. To isolate fungi from infected leaves, diseased leaves from various plants were collected using sterile scissors and placed in self-sealing bags using disposable gloves to prevent cross-contamination. Two diseased leaves were collected from each plant, and five plants were sampled. Collected diseased leaves were then cut into 5 × 5 mm tissue blocks at the junction of diseased and healthy leaves using sterile scissors. Subsequently, tissue blocks were soaked in 4% w/v sodium hypochlorite (NaClO) solution for 30 s, immersed in 75% v/v ethanol for 3 min, and rinsed with sterile distilled water (ddH_2_O) three times, with each rinse lasting more than 1 min. The treated leaves were then placed on potato dextrose agar (PDA) medium and incubated at 28°C for 5 days. Colonies grown on the medium were observed, and fungal colonies were purified by transferring single spores to obtain purified strains for further identification.

### 2.2 Morphological and molecular identification

Three representative fungal strains were examined microscopically, and the morphological characteristics of fungal conidia were observed using a Nikon Ni-D Microscopic System (Nikon Digital Sight 10). Sixty conidia were randomly selected, and their lengths and widths were measured and recorded. For molecular identification, genomic DNA of the isolated strains was extracted from hyphae growing on PDA medium at 28°C using Omega Fungal DNA Extraction Kit (CA, United States) following the manufacturer’s instructions. The extracted DNA was stored at −20°C. Partial sequences of the internal transcribed spacer (rDNA-ITS), beta-tubulin (*TUB*) gene, and glyceraldhyde-3-phosphate dehydrogenase (*G3PD*) gene were amplified by PCR using specific primers. The primers used were ITS1 (5′-TCCGTAGGTGAACCTGCGG-3′) and ITS4 (5′-GCTGCG TTCTTCATCGATGC-3′) (White et al., 1990), Bt2a-F (5′-GGTAACC AAATCGGTGCTGCTTTC-3′) and Bt2b-R (5′-ACCCTCAGTG TAGTGACCCTTGGC-3′) (Carbone and Kohn, 1999), and GD-F (5′-GCCGTCAACGACCCCTTCATTGA-3′) and GD-R (5′-GG GTGGAGTCGTACTTGAGCA-3′) (Templeton et al., 1992). PCR amplification was carried out using a Bio-Rad T100™ Thermal Cycler and a reaction system comprising the following for a total volume of 25 μL: 1 μL DNA template, 1 μL each of forward and reverse primers, 9.5 μL ddH_2_O, and 12.5 μL 2 × SanTaq PCR Mix. The PCR conditions consisted of initial denaturation at 94°C for 5 min, followed by 35 cycles of denaturation at 94°C for 30 s, annealing for 30 s at the corresponding temperatures (53°C for the ITS region, 55°C for the *TUB* gene, and 53.6°C for the *G3PD* gene), extension at 72°C for 45 s, and then a final extension at 72°C for 10 min. The PCR products obtained were sequenced by Qingke Bio (Chongqing) Technology Co., Ltd. The obtained sequences were compared and analyzed in GenBank. Subsequently, sequences were spliced according to ITS-*β-tubulin*-*G3PD*, and the phylogenetic tree was constructed using neighbor-joining clustering analysis in MEGA 7.0 software (Kumar et al., 2016). The obtained DNA sequences were uploaded to the National Center for Biotechnology Information.

### 2.3 Pathogenicity test

The mycelium disc inoculation method described by Chen Y. et al. (2021) were employed to confirm Koch’s postulates. Healthy walnut leaves were carefully selected for the experiment. Prior to inoculation, the leaves underwent a series of sterilization steps, including being rinsed with 75% alcohol for 1 min, washed with 0.4% sodium hypochlorite solution for 30 s, and then rinsed with sterile water three times (1 min each). Subsequently, the washed leaves were dried with sterile paper and gently scratched using a sterile inoculation needle. A 5 mm diameter mycelial plug of the representative isolate *D. segeticola* strain C27 was used to inoculate the sterilized leaves. Leaves inoculated with sterile PDA medium cakes were used as the control group. The inoculated leaves were kept moist by covering them with sterile absorbent cotton. The leaves were then incubated in a constant temperature incubator at 28°C, with a photoperiod of 16 h/8 h and relative humidity (RH) of 75%. Disease symptoms on the leaves were observed after 5 days of incubation. Three replicates were established for each treatment group to ensure the reliability and reproducibility of the results. The pathogenicity test was conducted and repeated three times.

### 2.4 Effect of temperature on mycelial growth, conidia production, and conidia germination rate

To investigate the impact of different temperatures on the colony diameter and conidia yield of the pathogenic fungal, a 5 mm diameter mycelial plug of pathogen *D. segeticola* C27 (7-day-old) was used to inoculate the center of sterile PDA culture medium and cultured in a constant temperature incubator at temperatures ranging from 5 to 35°C at 5°C intervals. Each treatment was repeated three times. Twelve days after inoculation, the growth diameter of hyphae was recorded using the cross method (Hu et al., 2022). Simultaneously, 10 agar-mycelium plugs containing hyphae were collected from the edge of pathogen colonies cultured for 12 days using a 5 mm sterile punch and transferred to sterile centrifuge tubes containing sterile water. The centrifugal tube containing the pathogenic fungal cakes was vigorously vortexed to dissolve the conidia from the pathogenic fungal mycelium in sterile water and filtered using sterile double-layer gauze. The number of conidia per square centimeter of the colony was counted using a hemocytometer and calculated using the following formula:


$$\mathrm{Conidia production}\;\left(\mathrm{conidia}/{\mathrm{cm}}^{2}\right)=X\times N\times 5\times 10^{4}/n\pi r^{2}$$

where X is the dilution fold, N is the number of conidia in five squares of the hemacytometer, n is the number of perforated agar–mycelium plugs, and r is the inner diameter of the perforator (Deng et al., 2012; Cao et al., 2022).

To determine the conidia germination rate, spores from the 12-day-old colony were washed with sterile water, and the conidia concentration was adjusted to 1 × 10^5^ conidia/mL using a hemocytometer. Subsequently, 0.2 mL of conidia suspension was added to 0.2 mL of potato broth medium (PDB) and cultured at different temperatures for 24 h. The number of germinated conidia was observed using a microscope, and the conidia germination rate was calculated accordingly.

### 2.5 Effect of photoperiod on mycelial growth, conidia production, and conidia germination rate

To investigate the effects of photoperiod on pathogen *D. segeticola* C27, the culture medium inoculated with pathogenic fungal (PDA for mycelial growth and conidia production, and PDB for conidia germination rate) was placed in a constant temperature incubator at 28°C. The following photoperiod settings were utilized: (a) 6 h light and 18 h dark; (b) 10 h light and 14 h dark; (c) 14 h light and 10 h dark; and (d) 18 h light and 6 h dark. Three replicates were established for each treatment group to ensure the reliability and reproducibility of the results.

### 2.6 Effect of different carbon and nitrogen sources on mycelium growth, conidia production, and conidia germination rate

The mycelium growth, conidia production, and conidia germination rate of *D. segeticola* C27 on various carbon and nitrogen sources were determined following methods adapted from prior research (Wu et al., 2023; Wang H. C. et al., 2023). Czapek’s medium served as the base medium, with sucrose or sodium nitrate substituted by different carbon or nitrogen sources of equal mass. Six carbon sources, namely glucose, maltose, soluble starch, D-fructose, lactose, and sucrose, were investigated alongside six nitrogen sources: NaNO_3_, (NH_4_)_2_SO_4_, beef extract, peptone, glycine, and urea. Representative fungal cakes measuring 5 mm in diameter were used to inoculate the center of Czapek’s agar plates containing different carbon and nitrogen sources on the periphery of PDA medium containing the pathogenic fungal. The plates were then cultured in a constant temperature incubator at 28°C for 12 days. Subsequently, the mycelium diameter, conidia production, and conidia germination rate were measured. Three replicates were established for each treatment group to ensure the reliability and reproducibility of the results.

### 2.7 Effect of humidity on conidia germination rate

To assess the impact of relative humidity on the conidia germination rate of *D. segeticola* C27, a suspension with a concentration of 1 × 10^5^ conidia/mL was applied to sterile glass slides and dried using sterile air in a sterile biosafety cabinet. Subsequently, the dried glass slides were placed in sterile Petri dishes, and the relative humidity levels (50, 60, 70, 80, 90, and 100%) were evaluated in culture at 28°C in the dark. After 24 h, conidia germination was observed using a microscope to calculate the conidia germination rate. Three replicates were established for each treatment group to ensure the reliability and reproducibility of the results.

### 2.8 *In vitro* antifungal activity of fungicides on mycelial growth

The antifungal activities of 14 chemical and 6 biological fungicides against *D. segeticola* C27 were assessed using the mycelium growth rate method (Xin et al., 2020). Different fungicides were dissolved in water or organic solvent, with specific solvents chosen according to the fungicide type (e.g., pyraclostrobin and benzalconazole were dissolved in ethyl alcohol; thiophanate-methyl and fluazinam were dissolved in acetone; pyraclostrobin tebuconazole, prochloraz, mancozeb, difenoconazole, flusilazole, dimetachlone, jingangmycin, metalaxyl hymexazol, kasugamycin, carvacrol, zhongshengmycin, ethylicin, tetramycin, thiram, zineb, and cymoxanil were dissolved in water). These solutions were then diluted (Table 1), added to PDA culture medium, and thoroughly mixed to create the treatment group. An equal amount of the respective solvent was added to the PDA culture medium as the control group (CK). The mixture was ventilated, cooled, and solidified. Subsequently, a 5 mm cake of the representative *D. segeticola* C27 strain was used to inoculate the center of fungicide culture media with different concentration gradients. The inoculated plates were placed in a constant temperature incubator at 28°C and 70% humidity for 12 days. The colony diameter (mm) was measured using the crisscross method. The percent inhibition of mycelial growth (PIMG) was calculated using the following formula (Usman et al., 2022):


$$\mathrm{PIMG}\left(\%\right)=\frac{C-T}{C-F}\times 100\%$$

where F is the diameter of the fungal plug, C is the radial growth diameter of the fungus in the control, and T is the radial growth diameter of the fungus in the treatment group.

**Table 1:** Concentrations of substances used for fungicide sensitivity assays and their China pesticide registration numbers (http://www.chinapesticide.org.cn).

Fungicide name	Registration number	Active ingredient	Manufacturer	Substance concentration (μg/mL)
Pyraclostrobin tebuconazole 30% SC	PD20172434	Pyraclostrobin 10% Tebuconazole 20%	Shaanxi Sunger Road Bio-science Co., Ltd. China	0.6, 0.15, 0.0375, 0.009375, 0.00234375
Pyraclostrobin 30% SC	PD20152488	Pyraclostrobin	Shenzhen Noposion Agrochemicals Co., Ltd. China	0.6, 0.15, 0.0375, 0.009375, 0.00234375
Prochloraz 45% ME	PD20081511	Prochloraz	Shenzhen Noposion Agrochemicals Co., Ltd. China	0.9, 0.45, 0.225, 0.1125, 0.05625
Mancozeb 430 g/L SC	PD20140953	Mancozeb	Guangdong Kefeng Technology Co., Ltd. China	0.43, 0.215, 0.1075, 0.05375, 0.026875
Difenoconazole 10% WG	PD20121603	Difenoconazole	Shandong Bainong Sida Biotechnology Co., Ltd. China	0.05, 0.0125, 0.003125, 0.00078125, 0.0001953125
Benzalconazole 50% EW	PD20131050	Benzalconazole	Jiangxi Buffett Chemical Co., Ltd. China	0.003125, 0.0015625, 0.00078125, 0.000390625, 0.001953125
Flusilazole 400 g/L EC	PD20085419	Flusilazole	Qingdao Hansen Bio-science Co., Ltd. China	0.1, 0.05, 0.025, 0.0125, 0.00625
Dimetachlone 40% WP	PD20150266	Dimetachlone	Jiangxi Heyi Chemical Co., Ltd. China	0.8, 0.4, 0.2, 0.1, 0.05
Jingangmycin 8% AS	PD93106-2	Jingangmycin	Zhejiang Tonglu Huifeng Biosciences Co., Ltd. China	163.84, 81.92, 40.96, 20.48, 10.24
Metalaxyl hymexazol 3% AS	PD20092691	Metalaxyl 0.5% Hymexazol 2.5%	Tianjin Luheng Chemical Co., Ltd. China	3.84, 1.92, 0.96, 0.48, 0.24
Kasugamycin 6% AS	PD20171513	Kasugamycin	Shanxi Xinyuan Huakang Chemical Co., Ltd. China	7.68, 3.84, 1.92, 0.96, 0.48
Carvacrol 5% AS	PD20171458	Carvacrol	Huazhi Hebei Biotechnology Co., Ltd. China	3.2, 1.6, 0.8, 0.4, 0.2
Zhongshengmycin 3% WP	PD20130210	Zhongshengmycin	Shenzhen Noposion Agrochemicals Co., Ltd. China	0.96, 0.48, 0.24, 0.12, 0.06
Ethylicin 80% EC	PD20101285	Ethylicin	Kaifeng Dadi Agrochemical Biotechnology Co., Ltd. China	0.4, 0.2, 0.1, 0.05, 0.025
Tetramycin 0.15% AS	PD20171878	Tetramycin	Liaoning Wkioc Bioengineering Co., Ltd. China	0.012, 0.006, 0.003, 0.0015, 0.00075
Thiophanate-Methyl 70% WP	PD20082098	Thiophanate-Methyl	Pilar (Shanghai)Biotech Co., Ltd. China	0.175, 0.0875, 0.04375, 0.021875, 0.0109375
Fluazinam 500 g/L SC	PD20151511	Fluazinam	Shandong Zouping Nongyao Co., Ltd. China	0.125, 0.00625, 0.03125, 0.015625, 0.0078125
Thiram 50% WP	PD20093058	Thiram	Shandong Bainong Star Biotechnology Co., Ltd. China	8, 4, 2, 1, 0.5
Zineb 80%WP	PD20100224	Zineb	Shandong Xinxing Pesticide Co., Ltd. China	1.6, 0.8, 0.4, 0.2, 0.1
Cymoxanil 52.5% WG	PD20060008	Cymoxanil	Shanghai Shengnong Pesticide Co., Ltd. China	16.8, 8.4, 4.2, 2.1, 1.05

EW, Emulsion Water; ME, Microemulsion; WG, Water-Dispersible Granule; WP, Wettable Powder; AS, Aqueous Solutions; EC, Emulsifiable Concentrates; SC, Suspension Concentrate.

The EC_50_ (concentration for 50% of the maximum effect) of different fungicides was calculated using toxicity regression analysis with DPS V7.05 software (Pasche et al., 2004). Three replicates were established for each treatment group to ensure the reliability and reproducibility of the results.

### 2.9 Data analysis

Data were analyzed using one-way analysis of variance and a least significant difference test in SPSS 27.0 software (IBM Corp., United States), and GraphPad Prism 9.0 software was used to create figures.

## 3 Results

### 3.1 Morphological characterization

**Figure 1:**
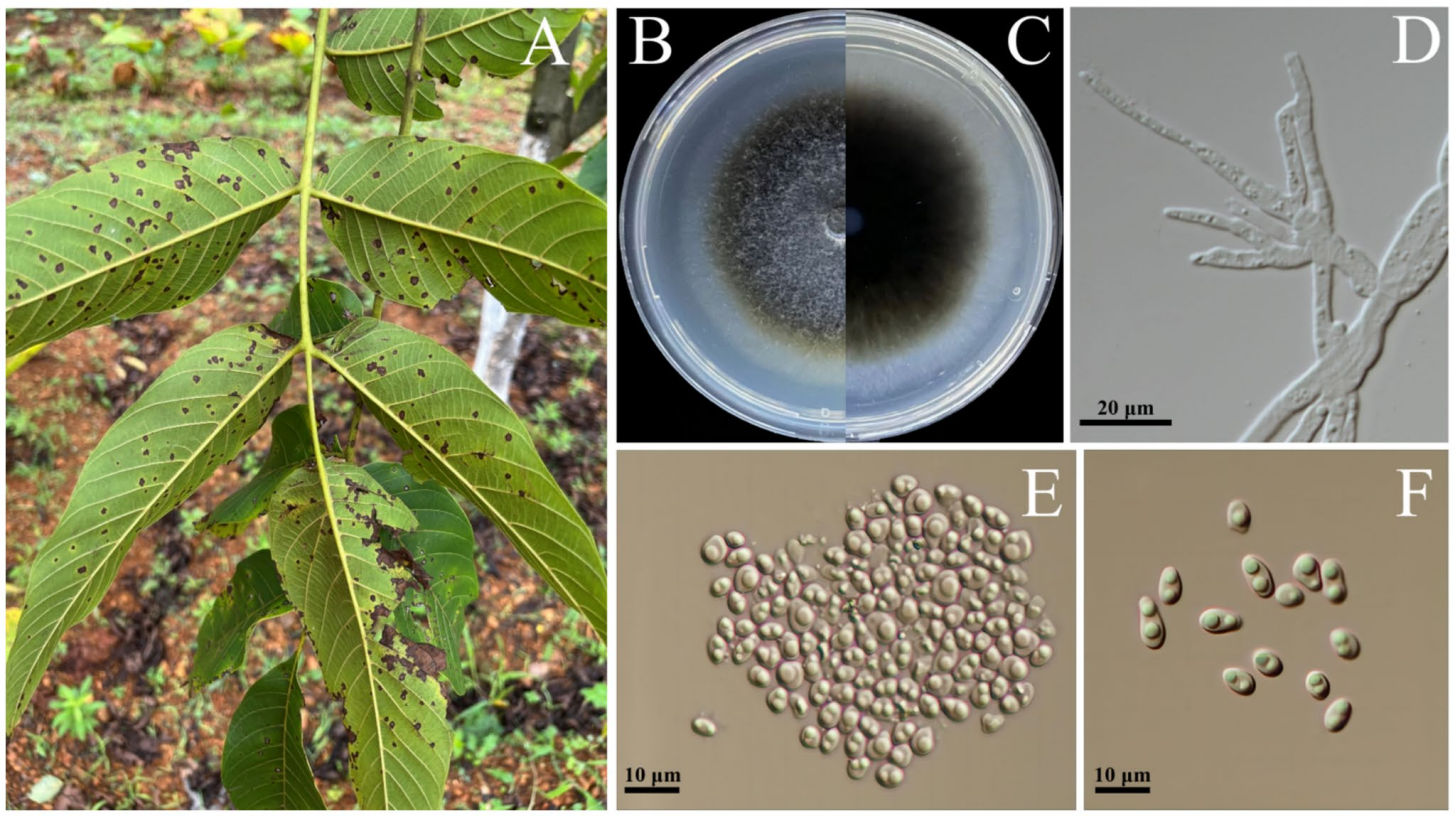
Leaf spot of walnut caused by *Didymella segeticola*. **(A)** Diseased leaf of walnut in field. **(B,C)** *D. segeticola* representative strain C27 colonies cultured on Potato Dextrose Agar medium for 12 days, front and back of Petri dish pictured. **(D)** Mycelium of *D. segeticola* C27 observed under the microscope. **(E,F)** Conidia of *D. segeticola* C27 observed under the microscope.

A total of 15 strains of *Didymella*-like fungi (Figure 1A) were successfully isolated from 10 diseased walnut leaves. Initially, the hyphae exhibited a grayish-white and the colonies had dense aerial hyphae. Over time, the colony became dark brown from the center outward, the colonies had woolly aerial hyphae, forming a distinct circular pattern, white to gray eventually (Figures 1B,C). Microscopic observation of three representative isolates revealed that the conidia were solitary, ellipsoidal, or obovate in shape, with rounded ends but varying sizes ranging from 4.2 to 6.8 μm × 2.5 to 3.1 μm (*n* = 50; Figures 1D,E). These morphological characteristics are consistent with the description of *Didymella* spp. (Chen et al., 2015).

### 3.2 Molecular characterizations

**Figure 2:**
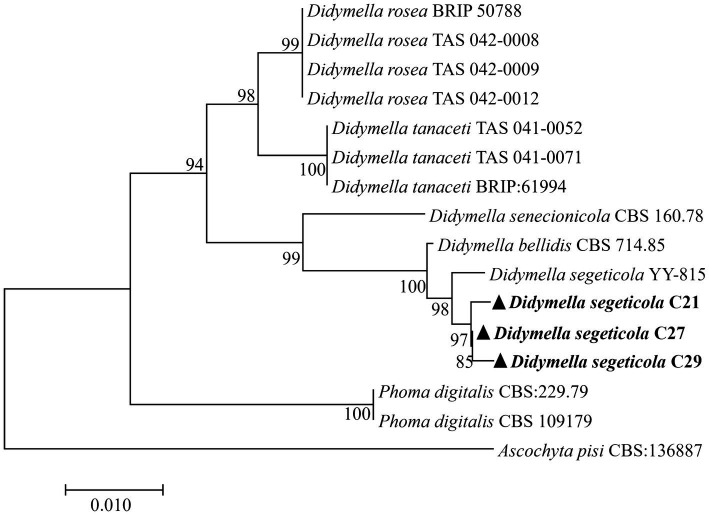
The neighbor-joining method (1,000 bootstrap iterations) was used to analyze concatenated sequences from the ITS region, *β-tubulin*, and *glyceraldhyde-3-phosphate dehydrogenase* genes from *Didymella segeticola* C21, *D. segeticola* C27 and *D. segeticola* C29 samples. *Ascochyta pisi* CBS:136887 was utilized as the outgroup. Bootstrap values are indicated next to the corresponding branches.

The obtained DNA sequences were compared with existing sequences available in GenBank through alignment. BLAST analysis of the sequenced fragments revealed the closest match to *D. segeticola* sequences, with high levels of identity: 99.44% for ITS (PP159078.1); 97.52% for *TUB* (KP330399.1); and 100% for *G3PD* (MZ844660.1). DNA sequences of representative strains were deposited in GenBank as follows: C21 (ITS: PP564883, TUB: PP592363, G3PD: PP592360), C27 (ITS: PP526746, TUB: PP592364, G3PD: PP592361), and C29 (ITS: PP565363, TUB: PP592365, G3PD: PP592362). Further, utilizing the sequences of the ITS region, *TUB,* and *G3PD* genes, a phylogenetic tree was constructed for the three representative strains using MEGA 7.0 software and the proximity method (Figure 2). The results showed that strains C21, C27, and C29 were clustered with *D*. *segeticola*.

### 3.3 Pathogenicity test

**Figure 3:**
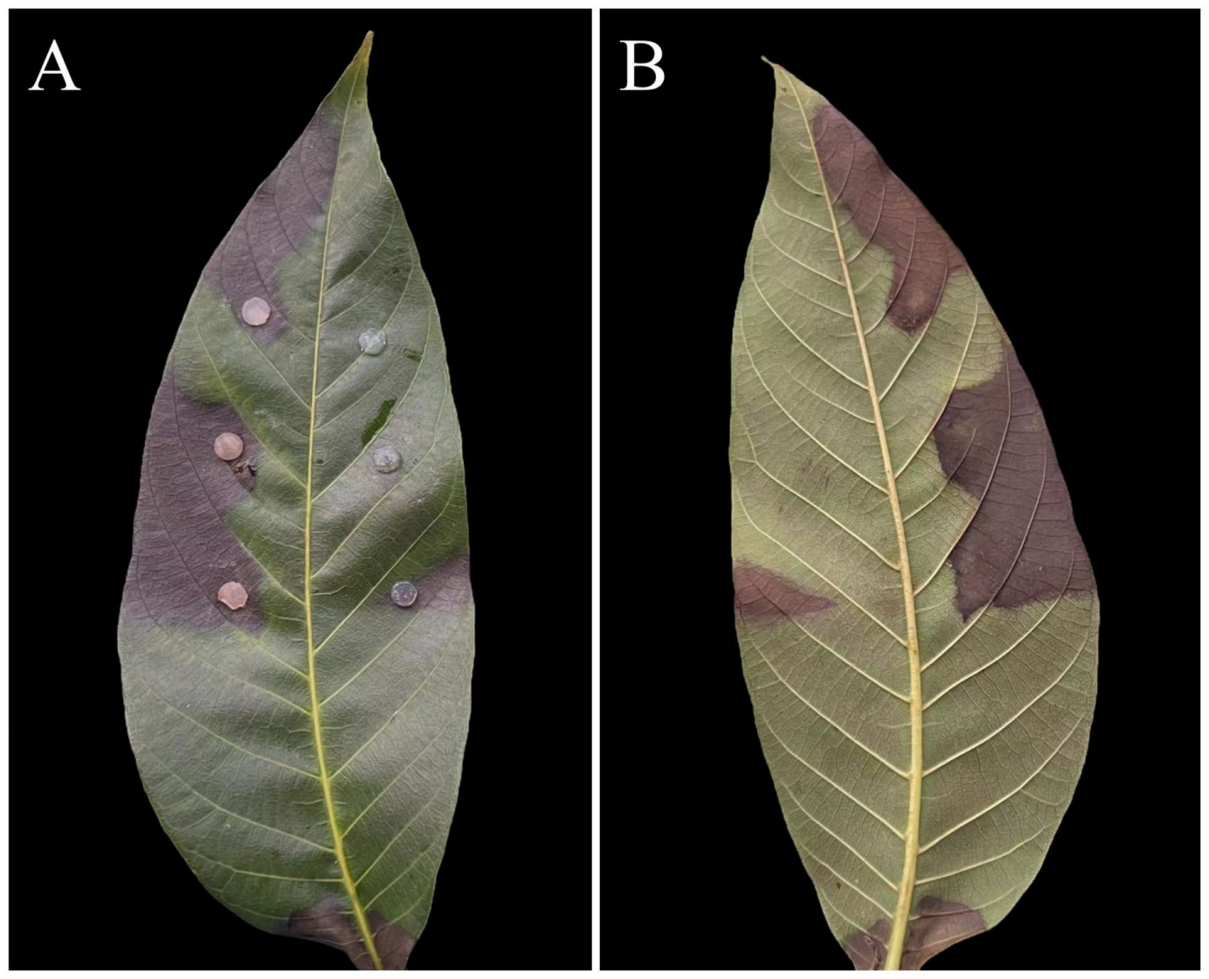
Symptoms of leaf spot caused by *Didymella segeticola* C27 used to inoculate walnut leaves: front side **(A)**; reverse side **(B)**.

Ten days post-inoculation, all of the healthy walnut leaves inoculated with the pathogen exhibited typical leaf spot symptoms (Figure 3), confirming the pathogenicity of the isolated strains. In contrast, the control leaves showed no disease symptoms. The pathogenic fungal were also successfully re-isolated from the inoculated leaves, confirming their role in causing the observed symptoms. Morphological characterization and molecular biology identification (utilizing ITS region, *TUB,* and *G3PD* gene sequences) confirmed that isolated strains C21, C27, and C29 belonged to the species *D*. *segeticola*. Subsequent experiments were conducted using pathogen C27 due to its consistent pathogenic behavior in the pathogenicity test.

### 3.4 Effects of temperature

**Figure 4:**
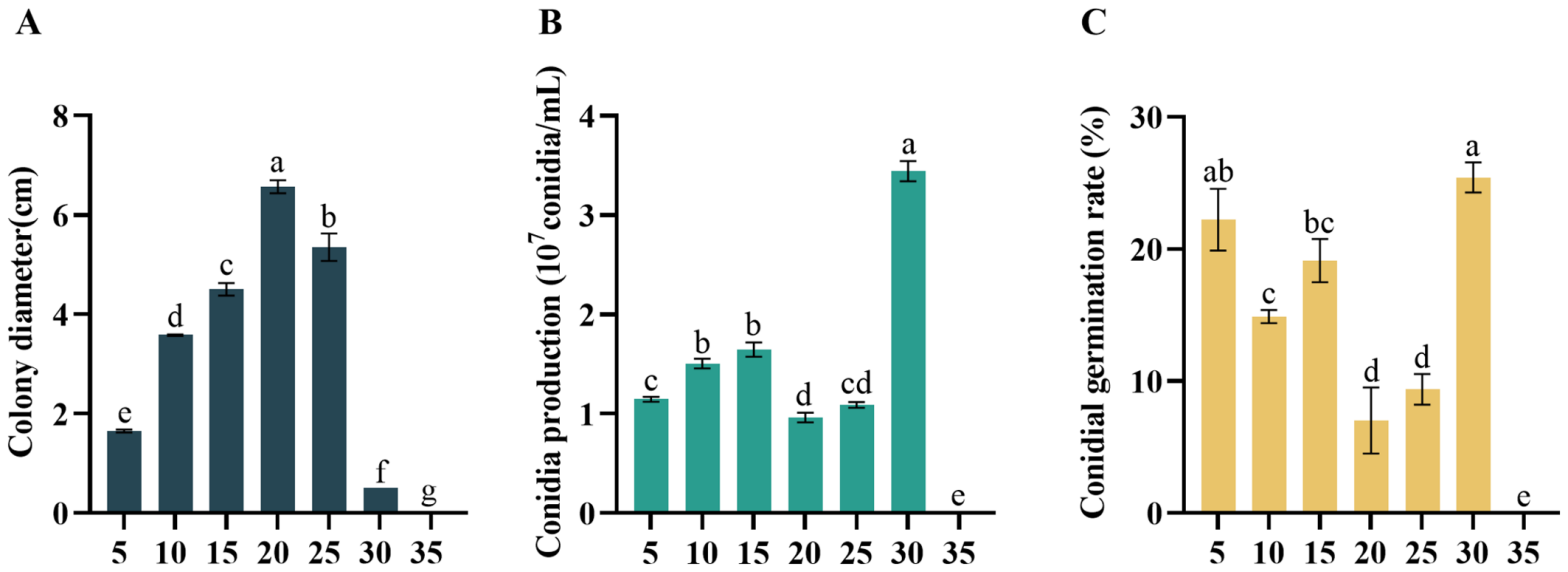
Effects of temperature (°C) on **(A)** mycelial growth, **(B)** conidia production, and **(C)** conidia germination rate of *Didymella segeticola* C27. Data are presented as the means ± standard error. Different lowercase letters indicate significant differences (*p* < 0.05).

*Didymella segeticola* C27 exhibited growth within a temperature range of 5–30°C. Notably, optimal growth conditions were observed at 20°C, with the highest mycelium growth rate recorded compared to other temperature treatments (Figure 4A). However, no mycelium or conidia growth was observed at 35°C. Regarding conidial production, the pathogen exhibited higher yields at 30°C compared to the other temperatures (Figure 4B). In addition, the conidia germination rate was highest at 30°C, exhibiting a significant difference compared to temperatures of 10, 15, 20, 25, and 35°C (Figure 4C).

### 3.5 Effects of photoperiod

**Figure 5:**
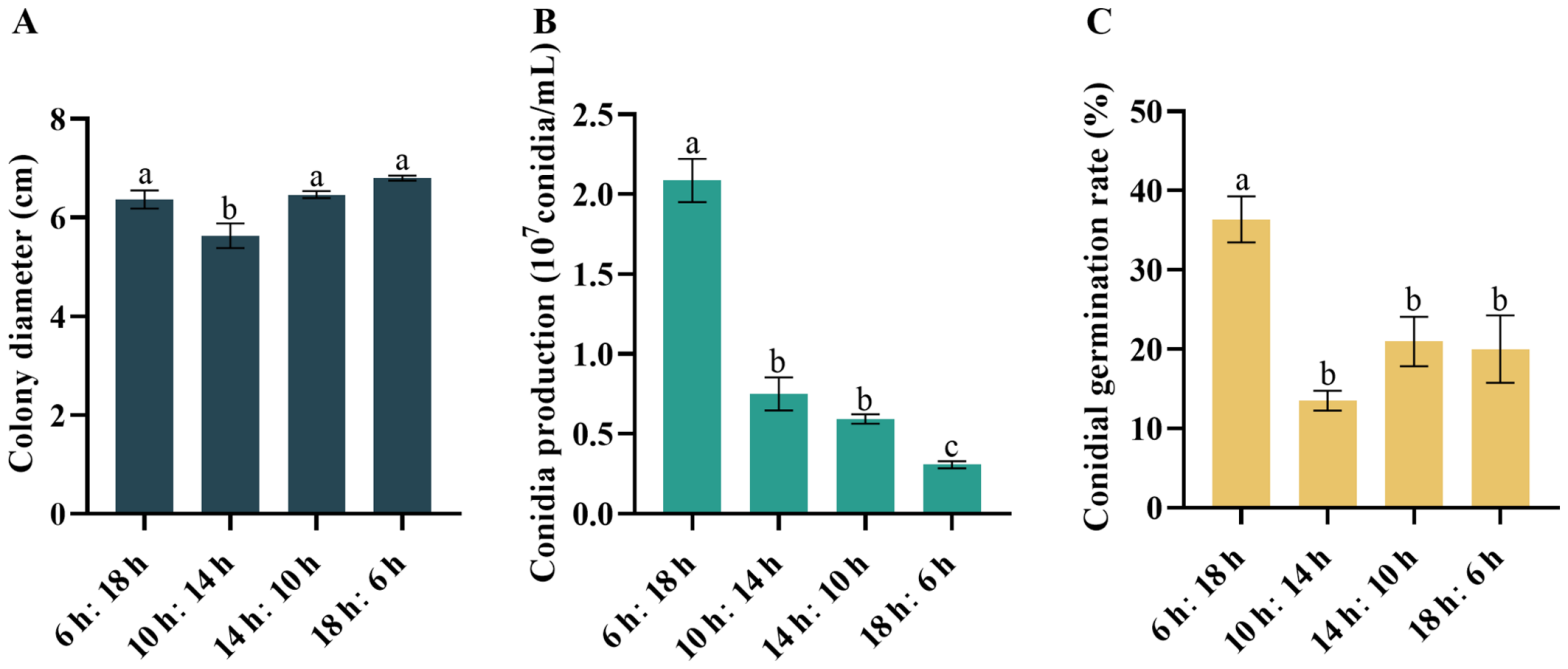
Effects of photoperiod on **(A)** mycelial growth, **(B)** conidia production, and **(C)** conidia germination rate of *Didymella segeticola* C27. Data are presented as the means ± standard error. Different lowercase letters indicate significant differences (*p* < 0.05).

Varying photoperiods had a discernible impact on the growth of *D*. *segeticola* C27. Specifically, the increase in mycelium growth was observed under photoperiods of 6/18 h, 14/10 h, and 18/6 h (Figure 5A). Notably, when the photoperiod was set to 6/18 h, the pathogen exhibited the highest number of conidia and the highest germination rate (Figures 5B,C). This photoperiod was particularly conducive to spore production and germination, and this effect was significantly different from the other treatment groups.

### 3.6 Effects of carbon resource

**Figure 6:**
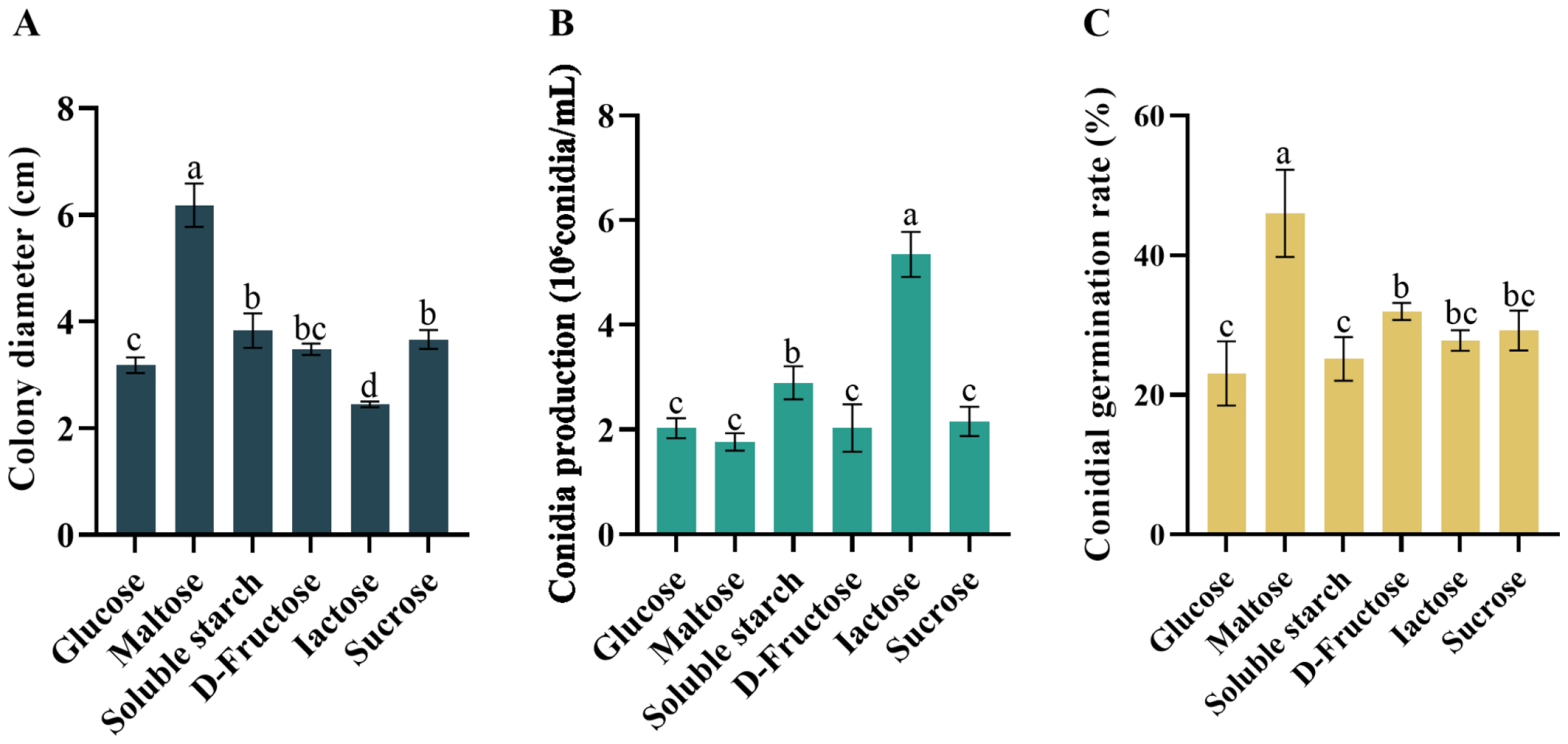
Effects of carbon resource on **(A)** mycelial growth, **(B)** conidia production, and **(C)** conidia germination rate of *Didymella segeticola* C27. Data are presented as the means ± standard error. Different lowercase letters indicate significant differences (*p* < 0.05).

All six of the carbon sources tested supported the growth, conidial sporulation, and conidia germination of the pathogenic fungal *D. segeticola* C27. However, maltose notably stood out as a carbon source, demonstrating significant promotion of both mycelial growth and conidia germination rates compared to other carbon sources (*p* < 0.05) (Figures 6A,C). Further, the utilization of lactose as a carbon source resulted in increased conidia production (Figure 6B).

### 3.7 Effects of nitrogen resource

**Figure 7:**
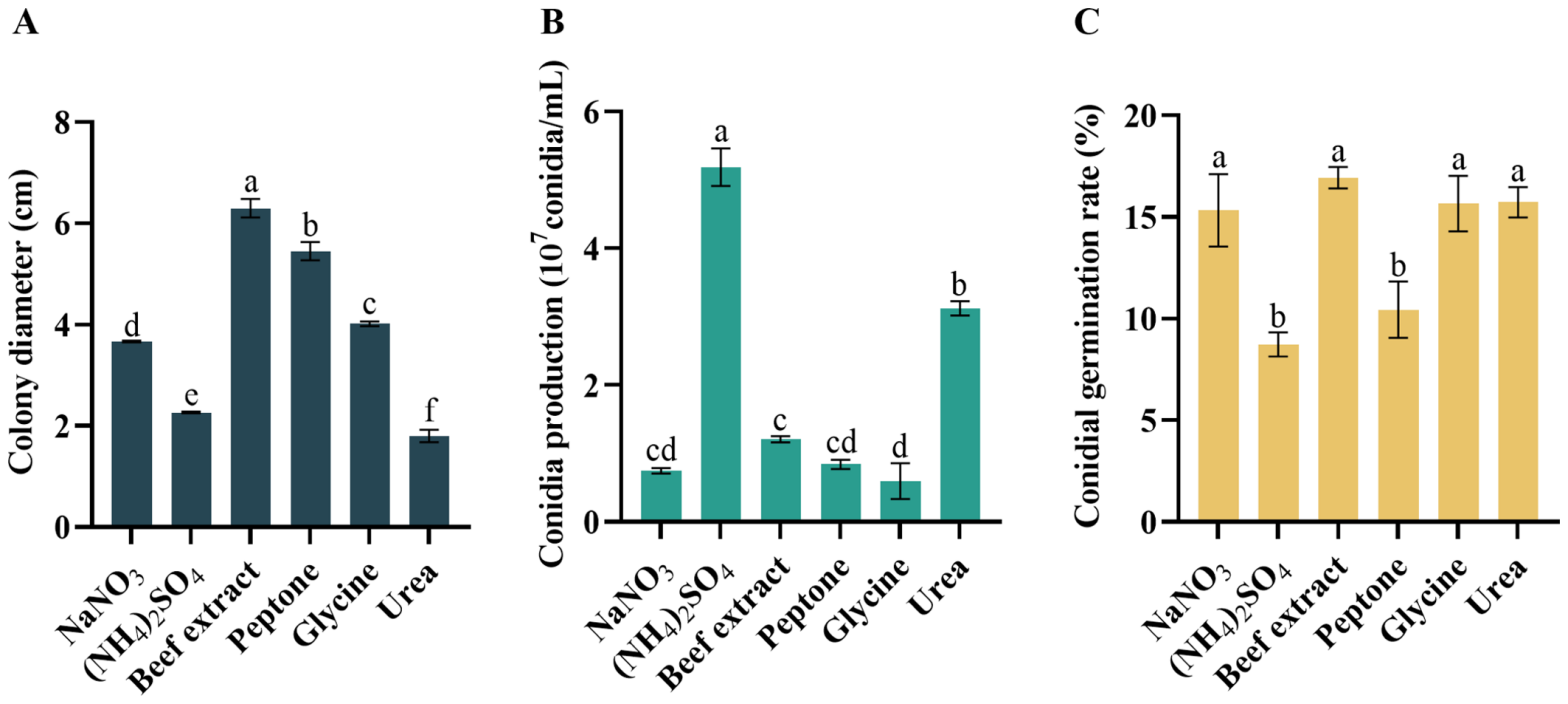
Effects of nitrogen resource on **(A)** mycelial growth, **(B)** conidia production, and **(C)** conidia germination rate of *Didymella segeticola* C27. Data are presented as the means ± standard error. Different lowercase letters indicate significant differences (*p* < 0.05).

The growth rate, conidia production, and conidia germination rate varied significantly under different nitrogen sources. Notably, the mycelium of *D. segeticola* C27 exhibited the fastest growth when beef extract was utilized as the nitrogen source, a difference that was significant compared to other nitrogen sources (*p* < 0.05) (Figure 7A). Further, the pathogen demonstrated enhanced conidia production when (NH_4_)_2_SO_4_ was employed as the nitrogen source (Figure 7B). In addition, the conidia germination rate was notably improved when the pathogen utilized NaNO_3_, beef extract, glycine, and urea as nitrogen sources, a difference that was statistically significant compared to (NH_4_)_2_SO_4_ and peptone nitrogen sources (*p* < 0.05) (Figure 7C).

### 3.8 Effect of different humidities

**Figure 8:**
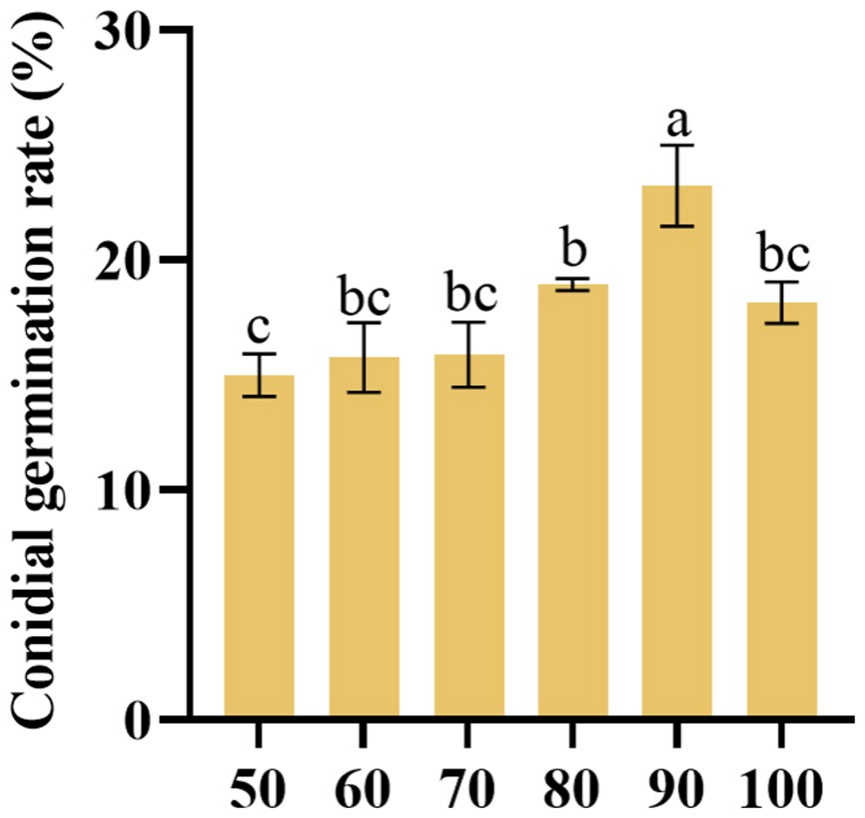
Effects of humidity (%) on the conidia germination rate of *Didymella segeticola* C27. Data are presented as the means ± standard error. Different lowercase letters indicate significant differences (*p* < 0.05).

The findings revealed that under 90% humidity, the germination rate of *D. segeticola* C27 was highest. This observed germination rate was significantly higher compared to other humidity conditions (*p* < 0.05) (Figure 8).

### 3.9 Fungicide assays

The sensitivity of the representative pathogen *D. segeticola* C27 to 20 selected fungicides was assessed (Table 2). All of the tested fungicides demonstrated inhibition of the mycelial growth of *D. segeticola* C27, as shown in Supplementary Figure S1. Among the 20 tested fungicides, the EC_50_ values of 16 fungicides were < 1 μg/mL. Difenoconazole had the best inhibitory effect, with an EC_50_ value of 0.0007 μg/mL, followed by tetramycin with an EC_50_ value of 0.0009 μg/mL. In addition, benzalconazole, flusilazole, pyraclostrobin tebuconazole, fluazinam, pyraclostrobin, prochloraz, ethylicin, mancozeb, thiophanate-methyl, dimetachlone, carvacrol, zineb, zhongshengmycin, and hymexazol also exhibited effective inhibitory effects against *D. segeticola* C27, with EC_50_ values ranging from 0.0036 to 0.6607 μg/mL. In contrast, kasugamycin, thiram, cymoxanil, kasugamycin, and jingangmycin displayed EC_50_ values larger than 1 μg/mL, with EC_50_ values of 1.3270, 1.6192, 7.2928, and 41.3217 μg/mL, respectively.

**Table 2:** Inhibitory effect of 20 fungicides on *Didymella segeticola* C27.

Fungicide	Toxic regression equation	EC_50_ (μg/mL)	r	95% Confidence intervals
Pyraclostrobin tebuconazole 30% SC	*y* = 6.3712 + 0.7333x	0.0135	0.9932	0.0083 ~ 0.0200
Pyraclostrobin 30% ME	*y* = 5.5140 + 0.2961x	0.0184	0.9436	0.0054 ~ 0.0438
Prochloraz 45% ME	*y* = 6.1164 + 0.7143x	0.0274	0.9979	0.0060 ~ 0.0546
Mancozeb 430 g/L SC	*y* = 6.2720 + 0.9651x	0.0481	0.9618	0.0305 ~ 0.0656
Difenoconazole 10% WG	*y* = 7.3798 + 0.7521x	0.0007	0.9901	0.0004 ~ 0.0011
Benzalconazole 50% EW	*y* = 7.3836 + 0.9767x	0.0036	0.9981	0.0023 ~ 0.0049
Flusilazole 400 g/L EC	*y* = 6.6864 + 0.8659x	0.0113	0.9964	0.0068 ~ 0.0158
Dimetachlone 40% WP	*y* = 5.8343 + 1.0220x	0.1527	0.9858	0.1132 ~ 0.1973
jingangmycin 8% AS	*y* = 3.4776 + 0.9420x	41.3217	0.9665	30.9868 ~ 55.1912
Hymexazol 3% AS	*y* = 5.2320 + 1.2890x	0.6607	0.9994	0.5145 ~ 0.8163
Kasugamycin 6% AS	*y* = 4.8265 + 1.4121x	1.3270	0.9965	1.0559 ~ 1.6138
Carvacrol 5% AS	*y* = 5.3359 + 0.6717x	0.3162	0.9756	0.1439 ~ 0.4882
Zhongshengmycin 3% WP	*y* = 5.5398 + 1.5216x	0.4418	0.9841	0.3616 ~ 0.5680
Ethylicin 80% EC	*y* = 6.5195 + 1.0949x	0.0409	0.9921	0.0268 ~ 0.0549
Tetramycin 0.15% AS	*y* = 7.6220 + 0.8540x	0.0009	0.9965	0.0004 ~ 0.0013
Thiophanate-Methyl 70% WP	*y* = 7.4139 + 2.0081x	0.0628	0.9619	0.0463 ~ 0.0939
Fluazinam 500 g/L SC	*y* = 6.6850 + 0.9594x	0.0175	0.9789	0.0119 ~ 0.0233
Thiram 50% WP	*y* = 4.7400 + 1.2423x	1.6192	0.9776	1.2770 ~ 2.0063
Zineb 80%WP	*y* = 5.4282 + 0.9372x	0.3492	0.9532	0.2572 ~ 0.4625
Cymoxanil 52.5% WG	*y* = 3.9861 + 1.1750x	7.2928	0.9175	4.5282 ~ 17.0558

EW, Emulsion Water; ME, Microemulsion; WG, Water-Dispersible Granule; WP, Wettable Powder; AS, Aqueous Solutions; EC, Emulsifiable Concentrates; SC, Suspension Concentrate.

## 4 Discussion

Walnut leaf spot stands out as a significant challenge in walnut cultivation, exerting detrimental effects on yield and quality (Wang F. H. et al., 2023). Prior investigations have underscored the havoc wreaked by *Didymella bryoniae*, inducing vine blight in melons and leading to substantial yield losses in 2004 (Sudisha et al., 2004). Similarly, *Phoma segeticola* was implicated in leaf spot disease in Tibetan thistle in 2015 (Chen et al., 2015), while *D. segeticola* was found to be a causal agent of leaf spot disease in tobacco, impairing tobacco quality (Guo et al., 2020). Further, its involvement in leaf spot disease in *Zanthoxylum bungeanum,* resulting in extensive leaf shedding and affecting seed yield and quality, was documented in 2022 (Yang et al., 2022). This study successfully identified the causal agent responsible for walnut leaf spot through comprehensive morphological, molecular, and pathogenicity assessments conducted in Hezhang County, Guizhou Province, China. To our knowledge, this is the first report of *D. segeticola* as the causal agent behind walnut leaf spot, providing crucial insights for policymakers to devise targeted control strategies.

It is pivotal to clarify the biological characteristics of plant pathogens for devising effective disease control strategies (Fan R. D. et al., 2023). Our study unveiled crucial insights into the biology of the walnut leaf spot fungus, *D. segeticola,* revealing its temperature and photoperiod preferences and its utilization of different carbon and nitrogen sources. Notably, we found that *D. segeticola* mycelium thrives within a temperature range of 5–30°C, with optimal growth observed between 20 and 25°C, aligning with prior findings in tobacco leaf spot disease (Wang H. C. et al., 2023). Further, maximal mycelial spore production was observed at 30°C, while the highest conidia germination rates were recorded at 5 and 30°C. Consistent with physiological analyses of tea leaf spot disease in Guizhou, we observed that *D. segeticola* failed to grow at 35°C (Cheng et al., 2022). Thus, for future walnut breeding, it is necessary to cultivate or select cultivars with high resistance to high temperature.

Photoperiod is an important environmental factor influencing the biological characteristics and pathogenesis of plant pathogens (Costa et al., 2021; Macioszek et al., 2021). In our study, we observed the fastest growth of pathogenic mycelium under photoperiods of 6:18 h (light: dark), 14:12 h, and 18:6 h, contrasting significantly with the 10:14 h photoperiod. Specifically, the 6:18 h photoperiod facilitated maximal spore production and germination rates, differing significantly from other photoperiod conditions. Hence, we suggest that the planting time of walnuts should be adjusted when planting walnuts in Guizhou, China.

Carbon source availability significantly impacts pathogen growth and spore germination. Our findings highlight maltose as the optimal carbon source, fostering the fastest mycelial growth and highest spore germination rates compared to other carbon sources, echoing results from research on tea spot caused by *D. segeticola* in Guizhou Province of China (Cheng et al., 2022). In addition, we observed maximal spore production when lactose served as the carbon source. Regarding nitrogen sources, beef extract emerged as the most conducive to mycelial growth, significantly differing from other nitrogen sources, in contrast to results observed in tea leaf spot caused by *Didymella bellidis* (Wu et al., 2023). Notably, (NH_4_)_2_SO_4_ supplementation resulted in the highest conidia production by pathogen C27, indicating its ability to thrive on diverse nitrogen sources. Moreover, NaNO_3_, beef extract, glycine, and urea resulted in the highest conidia germination rates, significantly differing from ammonium sulfate and peptone.

In addition, our study demonstrated that a humidity level of 90% facilitated the highest germination rate of pathogenic conidia, similar to the local humidity conditions in Bijie City, Guizhou Province, China, as reported by the China Meteorological Data Network. By monitoring the environmental humidity, an optimal drainage system can be established to prevent the soil from becoming excessively moist, and chemical agents under high humidity can also be utilized to effectively prevent and control diseases. These findings underscore the intricate interplay between environmental factors and the biology of *D. segeticola*, offering insights into tailored disease management strategies. The occurrences of plant disease caused by pathogenic fungi are closely related to the nutritional and environmental factors that when they providing feasible conditions for the pathogens to grow and spread (Tang et al., 2022). On the opposite, regulating nutritional and environmental conditions to establish the conditions that could adversely affect the growth of pathogens, may reduce the development and severity of plant disease (Fan K. et al., 2023). Therefore, these findings in our study on the new walnut leaf spot causal agent, *Didymella bryoniae*, could give new insights into the occurrences mechanisms of the disease, as well as provide biological and ecological basis for its effective control.

Chemical control remains the primary method for controlling fungal diseases, particularly those caused by the genus *Subspora* (Zhan et al., 2024). However, there are few studies on preventing and treating diseases caused by *D. segeticola*. Therefore, our study focused on screening fungicides against *D. segeticola* C27 *in vitro*. Among the 20 fungicides tested, comprising 14 chemical and 6 biological fungicides, the EC_50_ values of 3 fungicides were ≤ 0.01 μg/mL, while those of 16 fungicides were ≤ 1 μg/mL. The indoor toxicity test results revealed that difenoconazole was the most effective in controlling *D. segeticola*, with an EC_50_ value of 0.0007 μg/mL, deviating from prior research results, possibly due to differing hosts (Yang et al., 2022). Tetracycline followed with an EC_50_ value of 0.0009 μg/mL. Tetracycline is notable for its broad activity spectrum and low toxicity and is known to enhance plant disease resistance by inducing phenylalanine ammonia lyase (PAL), peroxidase (POD), and polyphenol oxidase (PPO) activities (Gao et al., 2018; Ma et al., 2018; Alqahtani et al., 2022). In addition, difenoconazole propiconazol demonstrated effective control, with an EC_50_ value of 0.0036 ug/mL. Notably, triazole fungicides effectively inhibited pathogenic fungal growth by interfering with ergosterol demethylation on the cell membrane, impeding membrane formation, and restraining pathogen growth (Vazquez et al., 2022). Conversely, jingangmycin exhibited weak inhibitory activity against *D. segeticola* C27, with an EC_50_ value of 41.3217 μg/mL. Nevertheless, prolonged and extensive use of chemical fungicides can adversely impact the ecological environment and food safety (Chen X. Y. L. et al., 2021). Hence, alternating between chemical and biological fungicides during control processes can mitigate pathogen resistance.

In summary, our study elucidated the biological characteristics, pathology, and effect fungicides for a walnut leaf spot pathogen in Guizhou Province of China. These findings could provide a foundation for the sustainable and effective management of walnut leaf spot caused by *D. segeticola* in Guizhou Province, facilitating informed decision-making for future disease control strategies.

## Data Availability

The datasets presented in this study can be found in online repositories. The names of the repository/repositories and accession number(s) can be found in the article/Supplementary material.
