# Supplementary material for: Biological characterization and in vitro fungicide screening of a new causal agent of walnut leaf spot in Guizhou Province, China
Source: Front Microbiol. 2024 Oct 9;15:1439487. doi: 10.3389/fmicb.2024.1439487 (PMC11500075; doi:10.3389/fmicb.2024.1439487)
Supplement: Supplementary file 1 [file Table_1.DOCX]

Supplementary Material

# Supplementary Data

*>Didymella segeticola* C21 ITS region. (accession number: PP564883)

AAGGATCATTACCTAGGAGTTGCGGGGCTTTGCCTGCCATCTCTTACCCATGTCTTTTGAGTACCTTACGTTTCCTCGGCGGGTCCGCCCGCCGACTGGACAATTTAAACCACTTGCAGTTGCAATCAGCGTCTGAAAAAACTTAATAGTTACAACTTTCAACAACGGATCTCTTGGTTCTGGCATCGATGAAGAACGCAGCGAAATGCGATAAGTAGTGTGAATTGCAGAATTCAGTGAATCATCGAATCTTTGAACGCACATTGCGCCCCTTGGTATTCCATGGGGCATGCCTGTTCGAGCGTCATTTGTACCTTCAAGCCTTGCTTGGTGTTGGGTGTTTGTCTCGCCTCTGCGCGTAGACTCGCCTCAAAACAATTGGCAGCCGGCGTATTGATTTCGGAGCGCAGTACATCTCGCGCTTTGCACTCAGAACGACGACGTCCAAAAGTACATTTTTACACTCTTGACCTCGGATCAGGTA

*>Didymella segeticola* C21 beta-tubulin (*TUB*) gene, partial cds. (accession number: PP592363)

GTAACCAAAACGGTGCTGCTTTCTGGCAGACCATCTCTGGCGAGCACGGCCTCGATGGCTCCGGTGTCTACAATGGCACCTCGGACCTTCAGCTCGAGCGCATGAACGTCTACTTCAACGAGGTACTAGAACCGACACGCTATCCTTAGACGGGCTGCGAGTGCTGACCTCTTCTAGGCCTCTGGCAACAAGTTCGTTCCCCGCGCCGTTCTCGTCGATTTGGAGCCCGGTACAATGGATGCTGTTCGCGCCGGCCCCTTCGGCCAGCTCTTCCGTCCGGATAACTTCGTCTTCGGCCAGTCTGGTGCTGGTAACAACTGGGCCAAGGGTCACTACACTGAGGGTA

*>Didymella segeticola* C21 glyceraldhyde-3-phosphate dehydrogenase (*G3PD*) gene, partial cds. (accession number: PP592360)

TGCCGTCAACGACCCCTTCATTGAGCCTCACTACGCTGTAAGCCTTCCATTGCTCGAAGTACCGGCTCATCGCGGATACAATGCTTCGAAGTTCTGCGCAGACGAGGACAAAAAGCTGACCAAACTTAGGCGTACATGCTCAAGTACGACTCCACCCA

*>Didymella segeticola* C27 ITS region. (accession number: PP526746)

ATCCTTTCCGTTAAAGGTACCTGCGGAAGGATCATTACCTAGAGTTGCGGGCTTTGCCTGCCATCTCTTACCCATGTCTTTTGAGTACCTTACGTTTCCTCGGCGGGTCCGCCCGCCGACTGGACAATTTAAACCACTTGCAGTTGCAATCAGCGTCTGAAAAAACTTAATAGTTACAACTTTCAACAACGGATCTCTTGGTTCTGGCATCGATGAAGAACGCAGCGAAATGCGATAAGTAGTGTGAATTGCAGAATTCAGTGAATCATCGAATCTTTGAACGCACATTGCGCCCCTTGGTATTCCATGGGGCATGCCTGTTCGAGCGTCATTTGTACCTTCAAGCCTTGCTTGGTGTTGGGTGTTTGTCTCGCCTCTGCGCGTAGACTCGCCTCAAAACAATTGGCAGCCGGCGTATTGATTTCGGAGCGCAGTACATCTCGCGCTTTGCACTCAGAACGACGACGTCCAAAAGTACATTTTTACACTCTTGACCTCGGATCAGGTAGGGATACCCGCTGAACTTAAGCATATCAAAAGGCGGGAGGAA

*>Didymella segeticola* C27 beta-tubulin (*TUB*) gene, partial cds. (accession number: PP592364)

GTAACCAAAACGGTGCTGCTTTCTGGCAGACCATCTCTGGCGAGCACGGCCTCGATGGCTCCGGTGTCTACAATGGCACCTCGGACCTTCAGCTCGAGCGCATGAACGTCTACTTCAACGAGGTACTAGAACCGACACGCTATCCTTAGACGGGCTGCGAGTGCTGACCTCTTCTAGGCCTCTGGCAACAAGTTCGTTCCCCGCGCCGTTCTCGTCGATTTGGAGCCCGGTACAATGGATGCTGTTCGCGCCGGCCCCTTCGGCCAGCTCTTCCGTCCGGATAACTTCGTCTTCGGCCAGTCTGGTGCTGGTAACAACTGGGCCAAGGGTCACTACACTGAGGGTA

*>Didymella segeticola* C27 glyceraldhyde-3-phosphate dehydrogenase (*G3PD*) gene, partial cds. (accession number: PP592361)

TGCCGTCAACGACCCCTTCATTGAGCCTCACTACGCTGTAAGCCTTCCATTGCTCGAAGTACCGGCTCATCGCGGATACAATGCTTCGAATCTGCGCAGACGAGGACAAAAAGCTGACCAAACTTAGGCGTACATGCTCAAGTACGACTCCACCCA

*>Didymella segeticola* C29 ITS region. (accession number: PP565363)

CCTTCCCGTAAAGGGGAACCTGCGGAAGGATCATTACCTAGAGTTGCGGGCTTTGCCTGCCATCTCTTACCCATGTCTTTTGAGTACCTTACGTTTCCTCGGCGGGTCCGCCCGCCGACTGGACAATTTAAACCACTTGCAGTTGCAATCAGCGTCTGAAAAAACTTAATAGTTACAACTTTCAACAACGGATCTCTTGGTTCTGGCATCGATGAAGAACGCAGCGAAATGCGATAAGTAGTGTGAATTGCAGAATTCAGTGAATCATCGAATCTTTGAACGCACATTGCGCCCCTTGGTATTCCATGGGGCATGCCTGTTCGAGCGTCATTTGTACCTTCAAGCCTTGCTTGGTGTTGGGTGTTTGTCTCGCCTCTGCGCGTAGACTCGCCTCAAAACAATTGGCAGCCGGCGTATTGATTTCGGAGCGCAGTACATCTCGCGCTTTGCACTCAGAACGACGACGTCCAAAAGTACATTTTTACACTCTTGACCTCGGATCAGGTAGGGATACCCGCTGAACTTAAGCATATCAAAAGCGGGGAGGAAA

*>Didymella segeticola C29 beta-tubulin (TUB) gene, partial cds. (accession number:* PP592365*)*

GTAACCAAAACGGTGCTGCTTTCTGGCAGACCATCTCTGGCGAGCACGGCCTCGATGGCTCCGGTGTCTACAATGGCACCTCGGACCTTCAGCTCGAGCGCATGAACGTCTACTTCAACGAGGTACTAGAACCGACACGCTATCCTTAGACGGGCTGCGAGTGCTGACCTCTTCTAGGCCTCTGGCAACAAGTTCGTTCCCCGCGCCGTTCTCGTCGATTTGGAGCCCGGTACAATGGATGCTGTTCGCGCCGGCCCCTTCGGCCAGCTCTTCCGTCCGGATAACTTCGTCTTCGGCCAGTCTGGTGCTGGTAACAACTGGGCCAAGGGTCACTACACT

*>Didymella segeticola* C29 glyceraldhyde-3-phosphate dehydrogenase (*G3PD*) gene, partial cds. (accession number: PP592362)

TGCCGTCAACGACCCCTTCATTGAGCCTCACTACGCTGTAAGCCTTCCATTGCTCGAAGTACCGGCTCATCAGCGGATACAATGCTTCAGAATCTGCGCAGACGAGGACAAAAAGCTGATCCAACTTAGGCGTACATGCTCAAGTACGACTCCACCCA

# Figure S1.TIFF


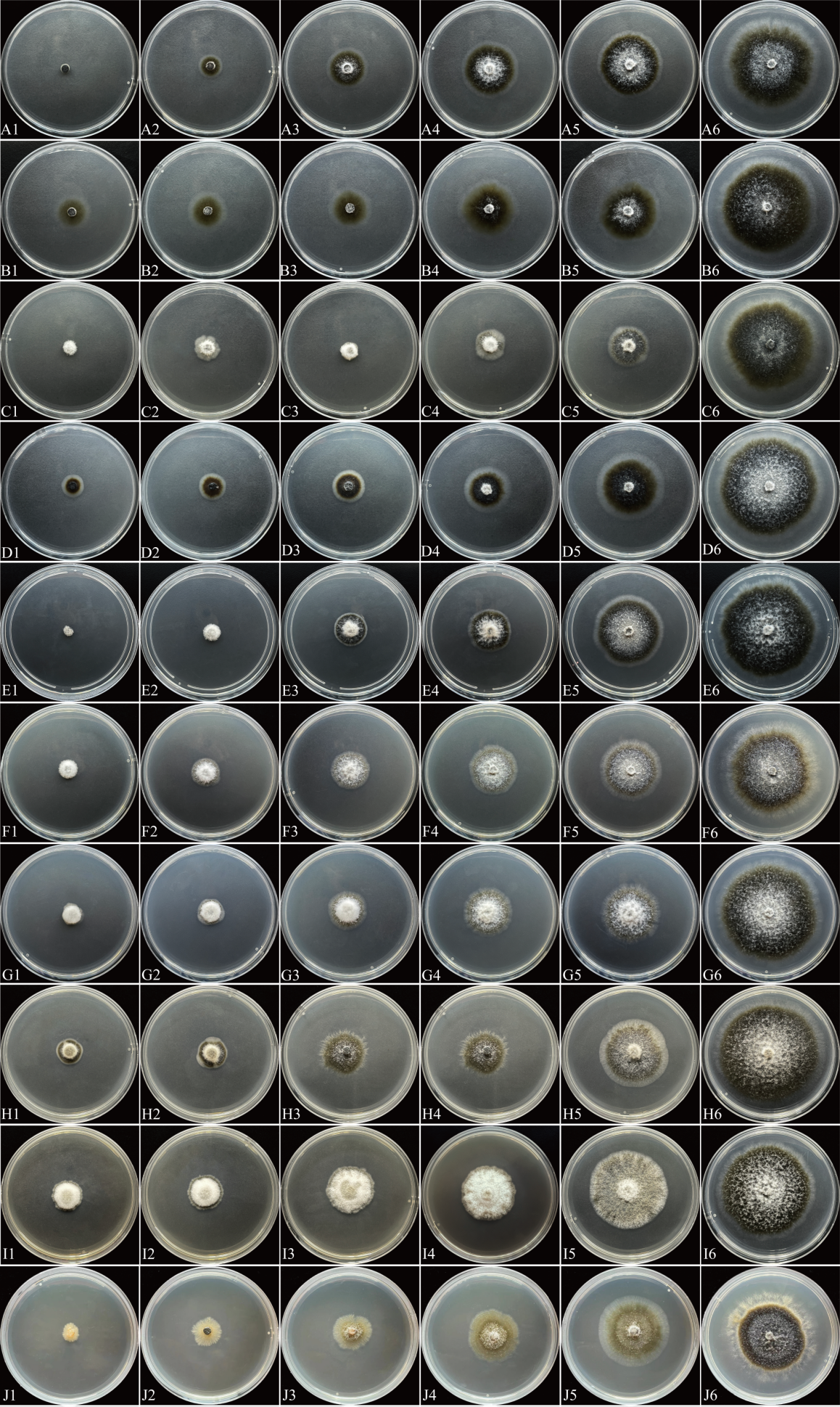


# Figure S1.TIFF (continue)


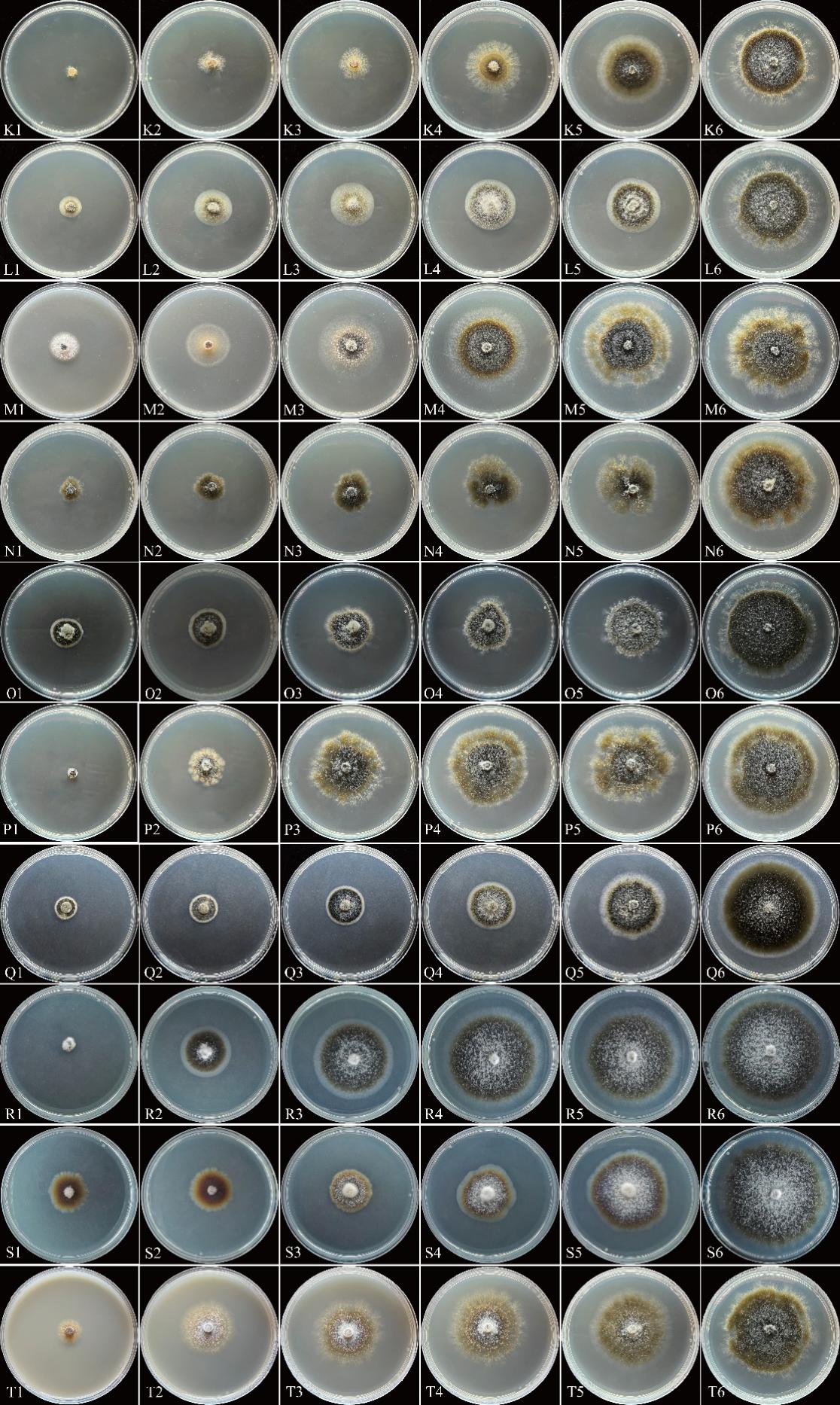


**FIGURE S1:** Mycelial growth of *Didymella segeticola* C27 on Potato Dextrose Agar plates incubated for 12 days in the absence (CK) or presence of different concentrations of pyraclostrobin tebuconazole (**A1**: 0.6 μg/mL, **A2**: 0.15 μg/mL, **A3**: 0.0375 μg/mL, **A4**: 0.009375 μg/mL, **A5**: 0.00234375 μg/mL, **A6**: CK), pyraclostrobin (**B1**: 0.6 μg/mL, **B2**: 0.15 μg/mL, **B3**: 0.0375 μg/mL, **B4**: 0.009375 μg/mL, **B5**: 0.00234375 μg/mL **B6**: CK), prochloraz (**C1**: 0.9 μg/mL, **C2**: 0.45 μg/mL, **C3**: 0.225 μg/mL, **C4**: 0.1125 μg/mL, **C5**: 0.05625 μg/mL, **C6**: CK), mancozeb (**D1**: 0.43 μg/mL, **D2**: 0.215 μg/mL, **D3**: 0.1075 μg/mL, **D4**: 0.05375 μg/mL, **D5**: 0.026875 μg/mL, **D6**: CK), difenoconazole (**E1**: 0.05 μg/mL, **E2**: 0.0125 μg/mL, **E3**: 0.003125 μg/mL, **E4**: 0.00078125 μg/mL, **E5**: 0.0001953125 μg/mL, **E6**: CK), benzalconazole (**F1**: 0.05 μg/mL, **F2**: 0.0125 μg/mL, **F3**: 0.003125 μg/mL, **F4**: 0.00078125 μg/mL, **F5**: 0.0001953125 μg/mL, **F6**: CK), flusilazole (**G1**: 0.1 μg/mL, **G2**: 0.05 μg/mL, **G3**: 0.025 μg/mL, **G4**: 0.0125 μg/mL, **G5**: 0.00625 μg/mL, **G6**: CK), dimetachlone (**H1**: 0.8 μg/mL, **H2**: 0.4 μg/mL, **H3**: 0.2 μg/mL, **H4**: 0.1 μg/mL, **H5**: 0.05 μg/mL, **H6**: CK), jingangmycin (**I1**: 163.84 μg/mL, **I2**: 81.92 μg/mL, **I3**: 40.96 μg/mL, **I4**: 20.48 μg/mL, **I5**: 10.24 μg/mL, **I6**: CK), metalaxyl hymexazol (**J1**: 3.84 μg/mL, **J2**: 1.92 μg/mL, **J3**: 0.96 μg/mL, **J4**: 0.48 μg/mL, **J5**: 0.24 μg/mL, **J6**: CK), kasugamycin (**K1**: 7.68 μg/mL, **K2**: 3.84 μg/mL, **K3**: 1.92 μg/mL, **K4**: 0.96 μg/mL, **K5**: 0.48 μg/mL, **K6**: CK), carvacrol (**L1**: 3.2 μg/mL, **L2**: 1.6 μg/mL, **L3**: 0.8 μg/mL, **L4**: 0.4 μg/mL, **L5**: 0.2 μg/mL, **L6**: CK), zhongshengmycin (**M1**: 0.96 μg/mL, **M2**: 0.48 μg/mL, **M3**: 0.24 μg/mL, **M4**: 0.12 μg/mL, **M5**: 0.06 μg/mL, **M6**: CK), ethylicin (**N1**: 0.4 μg/mL, **N2**: 0.2 μg/mL, **N3**: 0.1 μg/mL, **N4**: 0.05 μg/mL, **N5**: 0.025 μg/mL, **N6**: CK), tetramycin (**O1**: 0.012 μg/mL, **O2**: 0.006 μg/mL, **O3**: 0.003 μg/mL, **O4**: 0.0015 μg/mL, **O5**: 0.00075 μg/mL, **O6**: CK), thiophanate-methyl (**P1**: 0.175 μg/mL, **P2**: 0.0875 μg/mL, **P3**: 0.04375 μg/mL, **P4**: 0.021875 μg/mL, **P5**: 0.0109375 μg/mL, **P6**: CK), fluazinam (**Q1**: 0.125 μg/mL, **Q2**: 0.00625 μg/mL, **Q3**: 0.03125 μg/mL, **Q4**: 0.015625 μg/mL, **Q5**: 0.0078125 μg/mL, **Q6**: CK), thiram (**R1**: 8 μg/mL, **R2**: 4 μg/mL, **R3**: 2 μg/mL, **R4**: 1 μg/mL, **R5**: 0.5 μg/mL, **R6**: CK), zineb (**S1**: 1.6 μg/mL, **S2**: 0.8 μg/mL, **S3**: 0.4 μg/mL, **S4**: 0.2 μg/mL, **S5**: 0.1 μg/mL, **S6**: CK), and cymoxanil (**T1**: 16.8 μg/mL, **T2**: 8.4 μg/mL, **T3**: 4.2 μg/mL, **T4**: 2.1 μg/mL, **T5**: 1.05 μg/mL, **T6**: CK).
